# MindKind: A mixed-methods protocol for the feasibility of global digital mental health studies in young people

**DOI:** 10.12688/wellcomeopenres.17167.1

**Published:** 2021-10-15

**Authors:** The MindKind Consortium

**Keywords:** mental health, anxiety, depression, young people, mobile health study, qualitative research, data governance, engagement

## Abstract

**Background: **While an estimated 14-20% of young adults experience mental health conditions worldwide, the best strategies for prevention and management are not fully understood. The ubiquity of smartphone use among young people makes them excellent candidates for collecting data about lived experiences and their relationships to mental health. However, not much is known about the factors affecting young peoples’ willingness to share information about their mental health.

**Objective: **We aim to understand the data governance and engagement strategies influencing young peoples’ (aged 16-24) participation in app-based studies of mental health. We hypothesize that the willingness to participate in research is impacted by their ability to be involved in how their data is collected, shared, and used.

**Methods: **Here, we describe the MindKind Study, which employs mixed methods to understand the feasibility of global, smartphone-based studies of youth mental health. A pilot 12-week app-based substudy will query participants’ willingness to engage with remote mental health studies. Participants will be randomized into one of four different data governance models designed to understand their preferences, as well as the acceptability of models that allow them more or less control over how their data are accessed and used. Enrolees will receive one of two different engagement strategies. A companion qualitative study will employ a deliberative democracy approach to examine the preferences, concerns and expectations of young people, with respect to remote mental health research. We also detail our engagement with young people as co-researchers in this study. This pilot study is being conducted in India, South Africa and the United Kingdom.

**Conclusions: **This study is expected to generate new insights into the feasibility of, and best practices for, remote smartphone-based studies of mental health in youth and represents an important step toward understanding which approaches could help people better manage their mental health.

## Introduction

Anxiety and depression have a major impact on the lives of millions of individuals, particularly for adolescents and young people. For adolescents globally, mental health conditions constitute a major burden of disease, with one out of seven adolescents estimated to have mental health problems in 2019 ^1^. According to the World Health Organization, up to 20% of children and adolescents worldwide experience one or more mental health disorders with three-quarters of mental health related illnesses developing by their mid-20s ^2^. This burden has been exacerbated by the physical and mental impacts of the current Sars-Cov-2 pandemic ^3^. Despite the high prevalence rate, strategies for prevention and approaches to treating mental health in young people remain limited due to a myriad of factors, such as variable life circumstances and lived experience, lack of resources, shortage of trained health care providers, and social stigma associated with mental disorders.

To understand what kind of approaches will work for whom and why in relation to prevention, treatment and managing the ongoing difficulties of anxiety and depression in young people, it is important to gather data about the lived experience of young people with anxiety or depression. In order to study the variable impact of “lived experience” on disease trajectories and management, longitudinal data on lived experience must be collected across large study cohorts to detail the many aspects of that individual’s life, that may relate to or impact their mental health state, such as sleep, physical activity, social relationships, etc. Such data can be collected directly from individuals using digital technologies like smartphones. The ubiquity of smartphones and other modern technologies provides an opportunity for remote data collection of mental health in a convenient and non-intrusive manner. For longitudinal data especially, it can ensure rapid data collection with ease, in real life settings, without any travel or administrative barriers ^4^. Additionally, it opens avenues for large-scale data collection across borders that can yield data rich in the variability that could be key in understanding mental health.

The use of smartphones to capture participant-reported outcomes or e-diaries has become commonplace in health and mental health research ^5, 6^. In addition, mobile apps and digital sensors have been used in a number of research studies to monitor symptom variability ^7– 11^. Along with administering surveys, smartphone-based applications also enable the collection of data using evidence-based scales and instruments on factors that impact youth mental health along with passive data, such as the number of steps taken or screen time ^12^. Moreover, engaging youth through smartphones can have added benefits as they can be an effective medium to better understand young people's social, physical and emotional lives. However, the collection, processing, and use of such data, which is considered highly personal by many individuals, will need to be sensitive to the interests of the young people contributing their data. Very little is known about the factors influencing young peoples’ willingness to participate in such research, and across many domains, participation in remote studies tends to drop off quickly ^13^, so understanding the factors that would attract and maintain participation are important for the success of such research.

To this aim, we describe the MindKind study, a mixed-methods approach to understand the governance, technical and scientific feasibility of developing a global mental health database of digital data collected from young adults using smartphones. Our two-pronged approach pairs a pilot smartphone-based study with a qualitative study to understand the behaviour, concerns, and desires of young people with respect to mobile mental health research. We hypothesize that young peoples’ willingness to participate in research is impacted by their ability to be involved in how their data is collected, shared, and used. To that end, we will directly test a range of data governance models that promote participant-led open data practices ^14^. The overarching goals of the project are to: (a) prototype and test mechanisms to successfully empower young people to be involved in data management; (b) enable rapid and convenient data collection; (c) identify the governance model that young people support to share their data with mental health scientists; and (d) understand the factors affecting young peoples’ willingness to engage with and contribute to such a databank. This international effort is being piloted in three countries; India, South Africa and the United Kingdom (UK), which were chosen to have a range of economic, socio-cultural and regulatory landscapes. Throughout the project we centre our work on the voices of young people. We engage young adults with an interest in mental health as co-researchers and partners throughout the entire process, from design, prototyping, and testing of the study frameworks.

## Methods

### Ethics

MindKind has been approved by the relevant Institutional Review Boards and Ethics Boards in the U.S. (WIRB #20212067), UK (University of Cambridge - Department of Psychology Research Ethics Committee: Ref. PRE.2021.031 and University of Oxford: Ref. R73366/RE00), South Africa (Walter Sisulu University #029/2021 and the Department of Higher Education and Training), India (India Law Society #ILS/242/2021), and as of September 2021 is also currently under review by the Health Ministry Screening Committee (HMSC) in India.

### Study overview

MindKind is a feasibility study being conducted in India, South Africa, and the UK to capture the preferences and perspectives of youth from a set of diverse cultural backgrounds. The mental health data collected are not intended for analysis, but rather to understand the degree to which young adults are willing to share this information. The study’s aims are underpinned by the assumption that youth involvement in such a databank is essential to its success ^15^. In addition to the study design, we describe the roles of Youth Advisors and our Data Use Advisory Group in the development process. The study will employ mixed methods and consists of two substudies.

A quantitative substudy will query participants’ data governance preferences or acceptability, to understand how study participants would like their data governed and accessed. It will also collect demographic information and mental health indicators, in order to understand the willingness of participants to share information about their mental health on an ongoing basis. Participants will interact with a custom Android app to enable rapid and convenient data collection. Through a purpose-build app, we will examine such factors as consent to enrol, the types of data contributed, and duration of data contribution. Various engagement strategies will also be tested to determine how short- and long-term engagement may be impacted e.g., offering participants the choice of selecting which of the potential indicators of mental health--‘active ingredients’- they contribute to the databank. Here we define ‘active ingredients’ as factors which have been shown to influence mental health such as sleep, body movement/exercise, social connections, and positive activities ^16^.

The qualitative substudy will collect data from deliberative democracy sessions; a method that joins communities in discussions of complex ethical issues by providing education to inform discussion and engaging participants in dialogue leading to iterative consensus building ^17^, regarding youth databank governance preferences. It aims to (1) identify the consensus data governance model(s) for an open yet privacy-preserving global mental health databank, from the perspective of multinational young people; and (2) understand the concerns, hopes, and expectations of multinational youth for such a databank with regards to the return of value to youth participants and youth participation in databank governance.

### Interplay between quantitative and qualitative studies

Both studies aim to understand the best way to develop a global mental health databank for young people. The qualitative study generates data that can be implemented in future iterations of the app-based (quantitative) study. In its pilot phase, the quantitative study asks questions about engagement and acceptability within certain constraints, whereas the qualitative study asks about an ideal databank in an ideal scenario. The qualitative study exposes participants to options for data management and storage that are not feasible to ask of participants in the quantitative study. Additionally, whereas the quantitative study collects data that researchers can compare across regions, the qualitative study puts participants from different countries in direct dialogue with one another in the second round of deliberative sessions. This multinational session data will offer insights into why certain preferential differences may arise in the app-mediated study.

### Youth engagement

***Structure.*** This study leverages community-based participatory research approaches ^18^, which seek to involve community stakeholders and researchers as equal partners, to guide the engagement of young people with lived experience of mental health challenges. We define “lived experience” as self-reported experience with mental health challenges that cause a significant change in day-to-day functioning and contribution to community ^19^.

Young people are involved in this study in two ways: first as co-researchers ^20^, including study design, data collection, curation and analysis, and dissemination, and secondly as study participants (in the quantitative and qualitative substudies), whose preferences will inform the structure of the eventual mental health databank. By including young people as co-researchers and data contribution partners, we aim to: 1) centre our work from a social justice and health equity lens; 2) improve the quality and appropriateness of our study design, data collection, and data analysis; 3) empower young people as active agents for change in their communities; and 4) build capacity among young people around mental health and mental health research ^20, 21^.

Youth co-researchers have several formal roles within the project team:

*Professional youth advisors*: a professional youth advisor (PYA) was hired in each of the three countries to serve as a core member of the research team. PYAs were selected by the local site team based on their age (18–24 years old), lived experience with mental health challenges, and prior experience working on mental health-related, adolescent-focused, or community engagement projects and programs. As core members of the research team, PYAs contribute to help guide decisions and research directions. In addition to providing their own voice in research decisions, PYAs liaise between the country-specific Youth Panel Advisory Groups (YPAGs; see below) and the project teams, facilitating YPAG meetings, synthesizing panel feedback, providing recommendations to other project teams, and presenting their findings at monthly Steering Committee meetings. Each PYA led the recruitment for their respective YPAG and is responsible for conducting regular check-ins with YPAG members to ensure engagement throughout the project.

*Youth panel advisory groups*: single-country YPAGs have 12–15 members per site, ages 16–24 (UK) or 18–24 (India, South Africa), with lived experience of mental health challenges. YPAG members were purposefully recruited to ensure balance by gender, geographic regions, and educational background when possible. YPAGs meet twice per month via a video conferencing platform to discuss key aspects of the study design and data collection. Meetings are recorded and PYAs capture feedback in a study database after each meeting. YPAG members also participate in asynchronous virtual chats (e.g., via WhatsApp) which are particularly important for engaging YPAG members with limited data and/or internet access. YPAGs participate in small group projects and feedback rounds in between meetings as needed.

*International youth panel*: the international youth panel (IYP) has 14 members, with three to five representatives drawn from each of the YPAGs. The IPY meets virtually on a monthly basis and is a place for the youth across countries to get to know one another and to come to consensus around key decisions. IYP members were selected democratically by each YPAG. IYP meetings are recorded and feedback is captured within a study database.

*Global youth panel*: the global youth panel (GYP) has 15 youth representatives, ages 18–30, from each of the three country sites, as well as other high- and middle-income countries (e.g., United States, Canada, Kenya, Nigeria). Members were selected by the academic study team based on their past experience on youth panels and in advocacy groups for mental health issues among young people. The GYP meets virtually on a bi-monthly basis to provide high-level feedback on project decisions that could inform future testing and rollout of the MindKind study beyond our current three study locations. GYP meetings are recorded and feedback is captured within a study database.

***Feedback loops.*** Feedback from youth panels informs and guides project decision making as much as possible. Regardless of their ability to be implemented as part of this initial, feasibility study, all youth recommendations and decisions are documented and will be revisited for future, larger-scale studies based on this work.

Additionally, PYAs provide regular feedback on their roles in the project to the academic study team, allowing for meaningful review and revision of youth role descriptions and deliverables, identifying opportunities to improve equitable collaboration and devise study-specific metrics of successful youth engagement and study co-production.

### The data usability group

To advise on the scientific and analytical value of the future youth global mental health databank, we recruited and regularly convene a Data Usability Group (DUG) to provide their perspectives on scientific uses for a global mental health databank, governance models, data storage and accessibility, data use agreements, and researcher qualifications. The DUG has 20 researchers from seven countries (Australia, Brazil, India, Nigeria, South Africa, United Kingdom, and United States). DUG members are drawn from the fields of open science and data sharing, study teams associated with Wellcome ‘active ingredients’ commission ^16^, and adolescent mental health researchers and clinicians. The DUG meets virtually every three to six months, with more frequent asynchronous communication (e.g., follow up emails and surveys). Recommendations and feedback from the group is presented at Steering Committee meetings and consolidated into a database for tracking.

### MindKind study design

To participate in the MindKind study, youth participants must live in one of the participating countries and be legally able to provide consent (age 16–24 in the UK or 18–24 for India and South Africa). Youth are eligible if they can follow study instructions, read and understand English, and have access to an Android mobile phone (for quantitative substudy participation only).

***Quantitative substudy***

*Study design: governance models*

**Figure 1.**
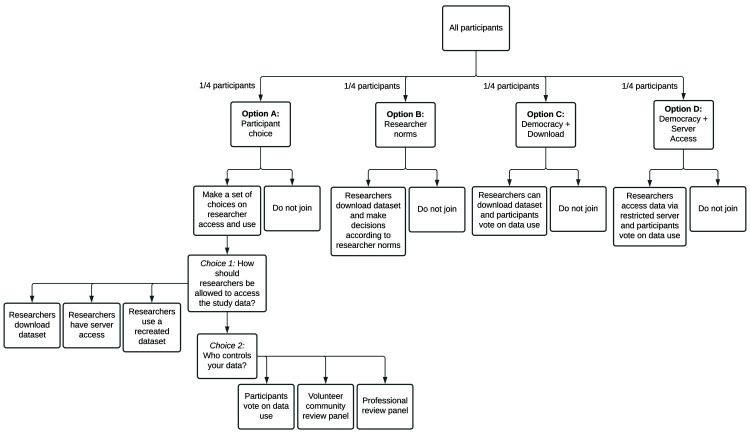
Governance study design. Potential quantitative substudy participants are randomly assigned to one of four consent models. Group A is designed to assess what practices are preferable to study participants. Groups B-D are designed to assess the acceptability of current standards to models that allow participants a greater voice and more data security.

The quantitative substudy is designed to assess the preference and acceptability of different data governance models and engagement patterns over time. Given the sensitivity of mental health data, we are seeking to understand whether prospective participants of a future global mental health databank have a preference for data governance models which give participants more control over who can access the data and for what purpose. We will also seek to understand whether these preferences impact enrolment. In order to assess these questions, youth participants will be randomized, in equal proportions, to one of four different governance experiences ( Figure 1), the first of which (Group A) assesses preference, with the remaining three (Groups B, C, D) addressing acceptability of various data governance models.

In order to assess the preference of young people with regards to data governance, participants randomized to Group A will be prompted to select how researchers are allowed to access their data, and who controls access to the data. The selection of these options was informed by the disparate preferences expressed by youth co-researchers versus the DUG.

Choice 1: How should researchers be allowed to access the study data?

1. Researchers should be allowed to download a copy.
2. Researchers should only be allowed to access the data in a secure server.
3. Researchers should only be allowed to see a recreated data set, not the real data. If researchers want to study the real data set, they have to ask the data steward to run their analysis for them. The steward only gives the researcher back the result, not the data.

Choice 2: Who controls the data?

1. Democracy: study participants who select this option get to vote on how the data is used, and the most popular terms are applied to all data regardless of how an individual votes. The results of the vote are shared with participants before data are shared. Any participant who disagrees with the vote may withdraw from the study. See Appendix 1 ^22^ ( *Extended data*) for voting questions.
2. Volunteer community review panel: participants selecting this option may choose to volunteer to serve as a data use request reviewer, taking one-week turns in this role on a rotating basis. Researchers will submit a statement telling the reviewers why they want to use the data. The reviewers will apply a set of criteria to decide yes or no. These criteria will be determined in advance by the whole group of volunteer reviewers.
3. Professional review panel: a paid panel will review data requests. This panel is a group of participants paid by the funder of the databank and may include research professionals (e.g., research ethics professionals). As above, researchers will have to submit a statement telling the reviewers why they want to use the data. The reviewers would decide yes or no, based on a set of criteria to which will be developed in advance by the group.

Participants randomized to Group A will be asked to select their data governance choices prior to consenting. Additionally, participants who select ‘Democracy’ for choice two will be asked to provide their preference on four questions about data terms of use (Appendix 1 ^22^, *Extended data*), which constitutes their democratic vote ( Figure 1).

In order to assess the acceptability of current governance standards relative to those that give participants a greater voice regarding how data are accessed and used, participants that are randomized to Groups B, C, or D will be presented with a pre-specified governance model. These three ‘acceptability’ experiences were selected by the research team to test (1) whether democratic determination of data terms improves enrolment over current researcher-driven norms, and (2) whether limiting data access to a restricted server further improves enrolment. Specifically, the three models are:

- Research norm (Group B): This option presents current researcher community norms for data use, whereby researchers will be able to download a copy of the data from the databank following strict data security rules. Data may be used, unrestricted, by both commercial and non-commercial researchers.
- Youth informed democracy with download (Group C): Study participants will vote as described in the Democracy Choice above. Under this model, researchers are allowed to download a copy of the data.
- Youth informed democracy without download (Group D): Study participants will vote as described in the Democracy Choice above. Under this data governance model, data may only be accessed via a restricted server.

In quantifying the difference in enrolment rates between participants in each group, we can assess whether democratic determination of access terms improves enrolment (Group C vs Group D), and whether restricting data download additionally improves enrolment (Group D vs Group C).

*Study design: engagement*

**Figure 2.**
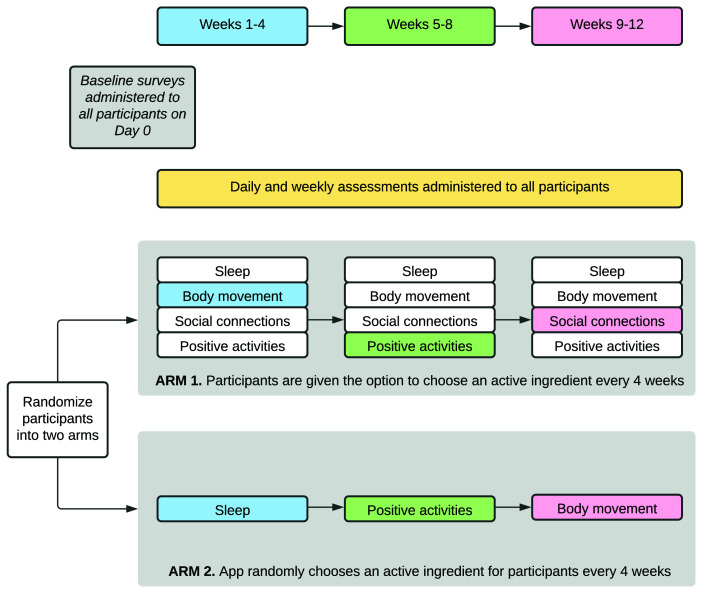
MindKind app study design. The 12-week study is composed of three, four-week rotations focusing on different topics influencing mental health (sleep, body movement, social connections and positive experiences). Participants will be randomized into one of two arms: the first of which allows participants to select their topics of focus and the second of which assigns the topics to which participants are exposed.

Following enrolment in the study, participants will use the study app to complete daily activities and surveys for the course of the 12-week study ( Figure 2). The study consists of questions about four domains (‘active ingredients’ (AIs)) which have been shown to influence mental health: sleep, body movement/exercise, social connections, and positive activities ^16^. Participants will focus on one of these domains in four-week rotations. For example, a participant may receive questions about body movement for weeks one to four, positive activities for weeks five to eight, and social connections for weeks nine to 12. See the “ *Surveys and data collection*” section for more details on the content of these domain surveys.

In order to understand whether the choice of survey domain impacts a participant’s short- or long-term engagement in the study, we will independently randomize participants into two different arms (in equal proportion). Participants in the first arm select their AI topic at the beginning of weeks one, five, and nine. Participants in the second arm are randomly assigned to their AIs for each of the three, four-week rotations ( Figure 2).

*Recruitment*

The quantitative substudy aims to recruit 4500 young people (1500 from each country) to download the study app, with a minimum of 10% of the 4500 young people recruited having lived experience of anxiety and depression. The sample size was selected based on expected engagement ^13^ in order to achieve a program goal of 100 participants per country completing the 12-week study.

Rolling recruitment will begin in the third quarter of 2021. Youth will be recruited in the following ways:

1. Through social media given limitations on in-person activities during the ongoing COVID-19 pandemic. Posts and advertisements will be placed on popular youth accessible social media platforms such as Instagram, Twitter, Facebook, LinkedIn, Reddit, and WhatsApp to reach young people in each of the three study countries.
2. To ensure that data collection is representative of a broad population in each country, the three sites will collaborate with intersectoral organisations (organisations working on sexual and reproductive health research, disabilities, sexuality, race/caste etc) with the help of emailers and posts.
3. Each of the sites will also reach out to partner (youth) organisations and spread the word within their networks along with using existing researcher contacts.
4. We will also recruit students at educational institutions (e.g., schools, colleges and universities) with the help of posters/flyers, and existing contacts between the site team and identified institutions.
5. Networks of young people will be tapped into by PYA and YPAG participants for snowball recruitment.

Recruitment materials such as social media posts, posters, flyers, videos etc, will be developed in English for all the three sites. Additionally, to reach a more diverse population, recruitment materials will be translated to other languages in South Africa (IsiXhosa and seSotho) and in India (Hindi, Marathi and Tamil). However, the study app will be available only in English.

Youth co-researchers have been heavily involved in the development of recruitment plans, providing feedback on the recruitment materials and helping disseminate the final materials to ensure that all recruitment materials are youth friendly and culturally appropriate.

Due to concerns about cellular data costs in South Africa, study participants in this country will be given a small stipend to subsidize their data plans in order to facilitate participation. Stipends of R150 will be paid per four weeks of participation in the quantitative substudy. Similarly, a small stipend pool exists for participants in India who find it difficult to pay for data. However, given the relatively low expense for cellular data here, the need is not expected to be widespread. No stipends are offered for participants of the quantitative substudy in the UK.

*Data collection*

**Figure 3.**
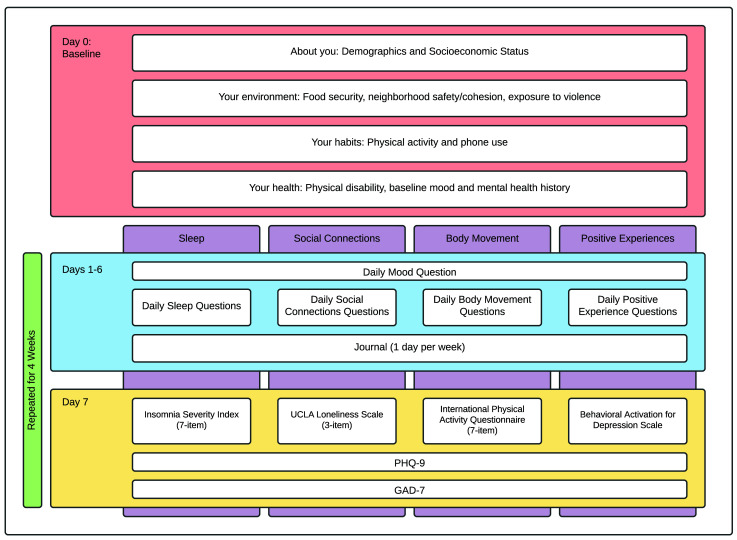
Baseline, daily, and weekly surveys. MindKind is a 12-week study consisting of a baseline survey followed by four-week rotations focusing on a single “active ingredient” (AI). On the seventh day of the week, a long survey is administered consisting of a standard instrument pertaining to the topic of the AI, as well as PHQ-9 and GAD-7. During the remaining days participants receive short questionnaires including a standard mood question and three to five AI-specific questions. Participants are prompted to journal on one of those days. At the beginning of weeks five and nine, a new AI is presented (Arm 2) or selected by the participant (Arm 1).

On enrolment, a baseline survey is administered to catalogue the participant’s background and experiences with mental health. For ease of administration, these are divided into four sections. ‘About you’ includes the topics demographics and socio-economic status (Appendix 3 ^23^, *Extended data*). ‘Your environment’ includes food security (USDA Food insecurity survey (six-item)) ^24^, neighbourhood safety and cohesion questions (PhenX Neighbourhood Safety, and PhenX Collective Efficacy) ^25^ and questions related to history of exposure to violence (Appendix 3 ^23^, *Extended data*). ‘Your habits’ includes questions pertaining to hobbies, physical activity and phone use habits (Appendix 3 ^23^, *Extended data*). ‘Your health’ includes questions pertaining to physical ability (WHODAS 2.0 (12-item) ^26^), depression (PHQ-9 ^27^), anxiety (GAD-7 ^28^), and history and management of mental health (Appendix 3 ^23^, *Extended data*). These baseline questionnaires are administered on study day zero ( Figure 3). On the following day, participants begin their first AI-rotation for weeks one to four. The second and third rotations occur weeks five to eight and nine to 12, respectively ( Figure 2).

**Surveys and active data collection:** The questions posed to participants throughout the study focus on the interplay between mood and four different AIs (sleep, social connections, body movement or physical activity, and positive activities) ( Figure 2– Figure 3). Study participants focus on one AI at a time, in four-week rotations. On days one to six of the week, participants are asked a standard mood question:

Pick the response that describes how you felt today:

◦ Worst ever
◦ Bad mood
◦ Average
◦ Good mood
◦ Best ever

along with a short (three to five item) AI-specific questionnaire (Appendix 2 ^29^, *Extended data*). They are also prompted to journal on an AI-specific or general topic on one of those days. On the seventh day, participants are asked to complete a long survey related to their AI topic (Insomnia Severity Index (seven-item) ^30^, UCLA Loneliness Scale (three-item) ^31^, International Physical Activity Questionnaire (seven-item) ^32, 33^, Behavioural Activation for Depression Scale ^34^ for the sleep, social connections, body movement and positive activities AIs, respectively), as well as PHQ-9 ^27^ and GAD-7 ^28^ to get a deeper understanding of their mood ( Figure 3).

**Passive data collection:** Participants can opt in to provide passive data collection about their phone activity and environment in order to understand their phone use habits. The statistics collected are daily screen time (a daily log of when the phone’s screen is unlocked), charging time (a log of when the phone is connected to its charger), battery statistics (a log of battery charge throughout the day via the Android ACTION_BATTERY_CHANGED call), data usage (hourly reporting of amount of data transmitted and received), ambient light as measured by the phone’s light sensor (sampled for ten seconds every five minutes in order to minimize battery consumption). No information will be collected that could violate a participant’s privacy, such as information about specific activities or apps used on the phone, call logs, or the content of messages. No identifiable location data will be collected.

**Technical implementation:** The study will be implemented in two separate pieces of technology. Eligibility checking, account creation and enrolment occurs via a website which has been optimized for mobile device experiences. Once a participant has enrolled, they are prompted to download the MindKind Study App from the Google Play store. The app requires an Android phone running Android 5.0 or higher. Participants use their phone number as a mechanism to create an account on the enrolment website, and to login to their account on the study app. Each time they request to login, a unique code sent to their phone via SMS validates their login.

Both applications will store data in the Bridge Server, a set of web services developed and operated by Sage Bionetworks. Bridge exposes a REST style web services API designed to allow collection and management of mobile health data from a variety of apps. This service has been used by Sage and other research organizations to support a variety of health studies on both the Android and iOS platforms. Bridge provides apps the ability to securely create accounts for participants, and record consent and other personal information separately from study data intended to be shared with research teams.

**Data management:** Data from both the enrolment website and study app will be sent to the Sage Bridge Server as json files. Coded study data, consisting of survey responses and passive data measurements, will be exported to Sage’s data sharing service, Synapse, for access by the study team ^35^. Synapse is a general-purpose data and analysis sharing service where members can work collaboratively, analyse data, share insights and have attributions and provenance of those insights to share with others. Synapse is developed and operated by Sage Bionetworks as a service to the health research community. Programmatic access to Synapse via R and Python Clients allows us to curate the json files from the surveys into tabular formats by survey type in order to allow for analysis.

*Data analysis plan*

All analyses described below will be performed within-site as well as across-site, adjusting for site. A sensitivity analysis will be performed, excluding South Africa, due to the potential confounding introduced by participant payment.

**Governance preference:** This analysis leverages the Group A ( Figure 1) selections for Option 1 and Option 2 to understand the degree to which study participants have a preference about how their data are accessed, both on who determines which investigators can access the data, and by what means they access the data. Outcome variables for this aspect of the substudy are the prospective participant’s (1) model choice for governing standard for the data and (2) model choice for researcher access to the data. The primary analysis will be a summarization of participant preference for each of the two questions. A chi-square test will be used to assess the statistical significance of the differences observed between the three options. A secondary set of analyses will assess the degree to which age, gender, and self-reported present or past mental health issues are associated with governance model preference, both globally and within-site. This analysis will also be performed with a multinomial logistic regression adjusting for these three variables.

**Governance acceptability:** This analysis leverages the enrolment rate for Groups B-D to understand the degree to which a governance model that improves usability and access by investigators, affects study enrolment over models that give participants more control (i.e. democratic determination of data terms) and offer better data security (i.e. secure server access only). The outcome variable for this aspect of the substudy is whether or not the prospective participant enrolled (yes/no). A logistic regression to test the effect of the governance model will be fit, adjusting for age, gender, self-reported present or past mental health issues, and site (global model only). The primary comparisons of interest are Group C vs Group B (democracy vs current norms) and Group D vs Group C (server access vs data download). A secondary analysis will test an interaction term between the model and self-reported mental health issues.

**Engagement:** The goal of this portion of the study is to understand participant engagement and compliance with the study protocol during the course of the 12-week study. We will measure participant engagement based on the following metrics: 1) study completion rates, i.e. proportion of participants that remain active in the study through 12 weeks as well as interim time points (e.g. four and eight weeks); 2) types of data (surveys and passive data streams) shared by participants and for how long; 3) total active tasks completed; 4) time to dropout (i.e. time point of the last task completed).

For study completion and interim participation, the binary outcome variables ‘participation at time X’ (yes/no) can be modelled using logistic regression to test the effect of the engagement model, adjusting for various socio-demographic factors (e.g. age, gender, self-reported history of mental health challenges), governance model (self-choice, or specific model presented) and site (global model only). For total task completion, the number of tasks completed by a participant will be modelled using Poisson Regression using the same independent variables as previously stated. For assessing the types of data shared by participants, we will compute the aggregate statistics on the number of unique data types shared by participants and for how long. Using a logistic regression model, we will assess the potential differences in data sharing aggregate statistics. Time to dropout can be analysed using a proportional hazards model with censoring at the study end. For each of these three analyses, we will also secondarily examine the effect of the governance model on engagement.

***Qualitative substudy***

*Study design*

We will employ a deliberative democracy approach for this study ^17^. Deliberative democracy is a method for community engagement in the complex ethical issues surrounding emerging technologies for which most people have not yet formed strong opinions. Deliberative democracy is a distinct qualitative research approach, differing from focus groups by the purposeful provision of educational intervention to enrich group discussion. Further, there is an emphasis on discussion leading to iterative revision of opinions as participants integrate new information and others’ perspectives.

We will conduct two rounds of deliberative democracy sessions. In the first round, up to ten cohorts of five to seven participants will be convened per country (approximately 150 total participants). A sample size of n=50 per site is characteristic of deliberative democracy studies ^36^. The individual group size of five to seven participants is characteristic of a typical small group in in-person studies ^36, 37^. Each will explore data governance models and voice their concerns and hopes regarding data governance for a global mental health databank for youth, identifying points of consensus and disagreement. Standard educational materials will be co-created and disseminated prior to the deliberative sessions to prepare participants. These materials may be disseminated as written or in other multi-media formats (e.g., audio, video). Participants will have the opportunity to ask the research team any questions prior to the sessions. Cohorts will meet virtually for up to two hours.

In the second round of deliberation, we will convene up to ten multinational cohorts; all participants in the multinational sessions will be drawn from the first round. Again, cohorts will meet for up to two hours in two 60-minute sessions. Each cohort includes six to eight participants: two to three participants from each country (60 total participants).

This arrangement of sessions allows participants to: 1) develop expertise over time both with content and with the deliberative approach; 2) appreciate the similarities and differences between country-specific and multinational perspectives on data governance; 3) reflect on the intersection of these topics with their own lived experience.

The quality of deliberative democracy sessions will be evaluated using criteria proposed by DeVries *et al.*, 2010 ^37^, including equal participation of all participants, respect for the opinions of others, willingness to adopt a social (rather than individualistic) perspective, and reasoned justification for one’s positions. These data will be gathered by the research team participant exit surveys.

*Co-creation*

YPAGs and the IYP engaged in co-design with the academic study team on the following topics: a) ensuring representation from marginalized and vulnerable groups in recruitment and participation; b) developing study materials including: informed consent, educational materials, and facilitator guides; c) identifying the challenges of virtual discussions and developing mitigation strategies to address equal engagement and power imbalances; d) planning for involving youth co-researchers in data analysis; e) dissemination of and access to study findings in a way that is engaging and inclusive of the people who have participated.

*Recruitment*

Participants will be recruited directly through YPAG members and existing volunteer rosters, existing researcher contacts, and snowball recruitment through these contacts. Further, social media (passive, active), direct email advertising, and posters may be used. Additionally, a single-time pop-up message in the MindKind app will recruit from young people participating in the quantitative substudy with a goal that half have previous exposure to the study. Young people with lived experience of anxiety and/or depression will be preferentially enrolled.

Deliberative sessions will be stratified by age and co-enrolment or lack of co-enrolment in the quantitative substudy. Youth will participate in sessions with others of similar age. For single country sessions, there will be up to three age cohorts (e.g., 16- to 17-year-olds in the UK; 18- to 20-year-olds in all countries; 21–24-year-olds in all countries). For multinational sessions, there will be at least two age cohorts (e.g., 16- to 20-year- olds; 21- to 24-year-olds). In single country sessions, we will aim for an equal distribution of participants across these two variables (i.e., age and co-enrolment in the quantitative substudy), but will have no fewer than one-third in each category, except for the UK age groups, which will have no fewer than one quarter of the total UK study population enrolled in any one age category. Additionally, we will seek a sample from a diversity of backgrounds in relation to gender, geographic location, socioeconomic status, educational attainment, ethnicity, religion, and first language. We will continue sampling participants until we have recruited a diverse set of individuals.

*Data collection and analysis*

There are two primary outputs of deliberative democracy processes: deliberative output and analytical output ^38^. Deliberative outputs are statements of consensus or disagreement that arise directly from discussants. These statements will be captured by the facilitator and will be ratified by discussants themselves before the end of their cohort session. We will employ the framework method ^39^ to capture analytical outputs regarding concerns, hopes, and expectations of discussants on return of value for participation in such a databank and the concerns, hopes, and expectations of discussants regarding youth participation. The framework approach is particularly useful in multi-disciplinary research teams that include lay people with less qualitative data analysis experience.

Discussion session data will be transcribed verbatim, de-identified, and checked for quality against audio recordings by the academic research team. De-identified transcripts will be uploaded to a cloud-based qualitative data analysis platform (NVivo). The academic research team, in collaboration with youth researchers, will code each transcript on this shared platform. The study team will identify *a priori* codes based on study goals, and emergent codes will be developed iteratively during the coding process. Coding discrepancies will be resolved through whole group discussion. Youth co-researchers will collaborate with the academic research team in the analysis process. All analytical outputs will be reviewed and ratified by the youth co-researchers and academic research team collectively.

## Discussion

The goal of this feasibility study is to further our understanding of the potential issues and challenges with developing a databank from remote, smartphone-based assessments of mental health in youth. Our findings will then inform the development of a larger global mental health databank and any future evaluations. Both the quantitative and qualitative study in the UK and South Africa are due to begin imminently, with the Indian site to follow once governmental approval is received.

We believe that the nature and extent of youth involvement in shaping an ambitious global mental health databank is uncommon in current research practices. The involvement of professional youth advisors in each of the sites, who in turn establish and run young people advisory groups (YPAGs) has been critical in ensuring that the study design, methods, engagement strategies, and app design are tailored to young people. While the development of the professional youth advisor role requires capacity building that must be undertaken for them to meaningfully engage with the study, the benefits of involving young people directly are manifold. To the best of our knowledge, this is the first time multi-site professional youth advisors have been engaged quite so closely in shaping the course of a project aimed at improving adolescent mental health globally.

### Challenges

This study benefits from site teams working in different contexts and is unusual in that two of the three study sites are from low-to-middle income countries (LMICs). However, these differences present some challenges. The sites have had to contextualize the data safety management and storage policies as per the sites and country-level laws and policies. In a digital feasibility study, the sites have had to ensure that concerns regarding data transfer to the main study site, privacy, and implications on the use of data collected were fully considered as a consortium along with the youth leads. Consistent, on-going consortium level meetings, consultations from experts on ethics, law and mental health researchers were some of the processes taken to ensure that the consortium is responsive to the ethical and cultural requirements.

This project has also faced planning and design challenges imposed by the COVID-19 pandemic. Many efforts initially envisioned as in-person, principally the youth panels, were transitioned to virtual venues. This has led to concerns about representation from marginalised and vulnerable groups, impacted by the inability to own a device to access the internet, gendered use of technology, and language barriers since the meetings were primarily held in English.

Integrating voices of young people also required strategies for protection against harm. This resulted in each site creating a safeguarding protocol that was contextually relevant for the group. Safeguarding included taking all reasonable steps to prevent any form of harm, abuse or neglect from occurring; protecting people's health, wellbeing and human rights; and further, taking reasonable steps to respond appropriately when harm or abuse does occur. As part of the process, the study was co-designed with young people and the consent forms and study material explained the purpose of the study in a simple manner.

We anticipate future challenges in implementing this study. For example, differences in ethical and institution specific procedural requirements that could potentially lead to delayed start dates for some study sites, which has implications on meeting the project timelines and milestones. Moreover, the varied timeline across sites has implications on equal participation from the study partners and youth engagement, such as formation of the youth panels or recruitment of the study team members across sites.

We also anticipate that recruiting 1500 young people for the quantitative study in the three sites may be challenging due to the size of this group proposed to be recruited and specific constraints in contexts. For example, there are lower numbers of young Android phone users in the UK. In contrast, the cost of accessing mobile data in South Africa may be prohibitive for many young people. We have made allowances for this by subsidizing cellular data costs; however, this introduces an incentive for participation ^13^ in the study and introduces potential confounding in the enrolment and engagement analyses.

With respect to the qualitative substudy, we anticipate that conducting deliberative democracy sessions on data governance models with young people who may not necessarily be familiar with the scientific literature may be another challenge. To mitigate this, we have prepared detailed recruitment strategies for each context and co-designed the educational material for deliberative democracy sessions along with youth advisors and YPAGs in each of the sites. Mock qualitative sessions were also held with YPAGs by the youth advisors to ensure the sessions planned were useful, accessible, and enjoyable for participants.

### Limitations

The primary potential limitation is that the views of those who use the MindKind app and participate in the deliberative democracy sessions in the UK, India, or South Africa may not be representative of young people as a whole in these countries or elsewhere. For example, due to the restrictions on the quantitative substudy, the study participants will include young adults that have access to the internet on an Android device and are able to interact with the digital platform in English. The sites made accommodations for providing access to data/internet for youth to engage with the study application, especially in low-middle income country settings. The qualitative substudy allows for flexibility on the language, on access to a device, and on the interaction with the study application, thereby allowing for a more diverse group of youth to opine on the study questions. This potential difference in representativeness is, however, expected and understanding these potential differences is part of this study. Moreover, the triangulation of data sources (app, group sessions, youth advisors’ feedback) will enable the consideration of similarities and differences between various sub-groups of young people. The study was not designed to assess mental health outcomes, and therefore any analyses of the data pertaining to these outcomes must be interpreted with caution.

## Data availability

### Underlying data

No underlying data are associated with this article.

### Extended data

Synapse: MindKind Databank

- **Appendix 1: (Democratic choice voting options, PDF format)** ^22^
https://doi.org/10.7303/syn26067677.1
- **Appendix 2: (Daily AI questions, PDF format)** ^29^
https://doi.org/10.7303/syn26067678.1
- **Appendix 3: (Eligibility and Baseline questionnaires, PDF format)** ^23^
https://doi.org/10.7303/syn26067679.1

Data are available under the terms of the Creative Commons 1.0 “Universal” ( CC0 1.0 public domain dedication)

## The MindKind Consortium

Faith Oluwasemilore Adeyemi ^1, 2^, Patricia A. Areán ^3^, Emily Bampton ^4^, Ljubomir Bradic ^5^, Anne-Marie Burn ^1^, Emma Carey ^1^, Sonia P. Carlson ^5^, Pamela Y. Collins ^3, 6^, Tessa Concepcion ^6^, Meera Damji ^7^, Megan Doerr ^5^, Julia C. Dunbar ^5^, Mina Fazel ^4, 8^, Blossom Fernandes ^4^, Gillian Finchilescu ^9^, Tamsin Jane Ford ^1, 10^, Melvyn Freeman ^11, 12^, Isabell R. Griffith Fillipo ^3, 13^, Jay Hodgson ^5^, Jasmine Kalha ^7^, Minal Karani ^7^, Michael R. Kellen ^5^, Christopher G. Kemp ^14^, Simthembile Lindani ^15^, Lara M. Mangravite ^5^, Carly Marten ^5^, Felicia Mata-Greve ^3^, Emily Moore ^5^, Erin Mounts ^5^, Lakshmi Neelakantan ^4^, Larsson Omberg ^5^, Lisa Pasquale ^5^, Soumitra Pathare ^7^, Swetha Ranganathan ^7^, Nichole Sams ^3^, Erin Scanlan ^5^, Himani Shah ^7^, Sotirios Short ^11^, Refiloe Sibisi ^11^, Solveig K. Sieberts ^5^, *, Stockard Simon ^5^, Sushmita Sumant ^7^, Christine Suver ^5^, Chandre Van Vught ^11^, Jennifer Velloza ^6^, Zukiswa Zingela ^16^

^1 ^Department of Psychiatry, University of Cambridge, Cambridge, United Kingdom

^2 ^Department of Psychology, University of Bath, United Kingdom

^3 ^Department of Psychiatry & Behavioral Sciences, University of Washington, Seattle, Washington, United States

^4 ^Department of Psychiatry, University of Oxford, Oxford, United Kingdom

^5 ^Sage Bionetworks, Seattle, Washington, United States

^6 ^Department of Global Health, University of Washington, Seattle, Washington, United States

^7 ^Centre for Mental Health Law and Policy, Indian Law Society, Pune, Maharashtra, India

^8 ^The Oxford Psychological Medicine Centre, Oxford University Hospitals NHS Foundation Trust, Oxford, United Kingdom

^9 ^Department of Psychology, University of the Witwatersrand, Johannesburg, Gauteng, South Africa

^10 ^Cambridge and Peterborough NHS Foundation Trust, Cambridge, UK

^11 ^Higher Health, Higher Education and Training: Health, Wellness, and Development Centre, Pretoria, Gauteng, South Africa

^12 ^Department of Psychology, University of Stellenbosch, Stellenbosch, Western Cape, South Africa

^13 ^CREATIV Lab, University of Washington, Seattle, Washington, United States

^14 ^Department of International Health, Johns Hopkins University, Baltimore, Maryland, United States

^15 ^Department of Psychiatry and Behavioural Science, Walter Sisulu University, Mthatha, Eastern Cape, South Africa

^16 ^Office of The Executive Dean, Faculty of Health Sciences, Nelson Mandela University, Gqeberha, Eastern Cape, South Africa.

* Corresponding author: Solveig Sieberts ( solly.sieberts@sagebase.org)

## References

[1] GBD 2019 Diseases and Injuries Collaborators: Global burden of 369 diseases and injuries in 204 countries and territories, 1990-2019: a systematic analysis for the Global Burden of Disease Study 2019. *Lancet.* 2020;396(10258):1204–1222. 10.1016/S0140-6736(20)30925-9 33069326 PMC7567026

[2] Kessler RC Angermeyer M Anthony JC : Lifetime prevalence and age-of-onset distributions of mental disorders in the World Health Organization's World Mental Health Survey Initiative. *World Psychiatry.* 2007;6(3):168–176. 18188442 PMC2174588

[3] Singh S Roy D Sinha K : Impact of COVID-19 and lockdown on mental health of children and adolescents: A narrative review with recommendations. *Psychiatry Res.* 2020;293:113429. 10.1016/j.psychres.2020.113429 32882598 PMC7444649

[4] Fischer F Kleen S : Possibilities, Problems, and Perspectives of Data Collection by Mobile Apps in Longitudinal Epidemiological Studies: Scoping Review. *J Med Internet Res.* 2021;23(1):e17691. 10.2196/17691 33480850 PMC7864774

[5] Golan D Sagiv S Glass-Marmor L : Mobile-phone-based e-diary derived patient reported outcomes: Association with clinical disease activity, psychological status and quality of life of patients with multiple sclerosis. *PLoS One.* 2021;16(5):e0250647. 10.1371/journal.pone.0250647 33951061 PMC8099126

[6] Rijsbergen M Niemeyer-van der Kolk T Rijneveld R : Mobile e-diary application facilitates the monitoring of patient-reported outcomes and a high treatment adherence for clinical trials in dermatology. *J Eur Acad Dermatol Venereol.* 2020;34(3):633–639. 10.1111/jdv.15872 31419338 PMC7064941

[7] Bot BM Suver C Neto EC : The mPower study, Parkinson disease mobile data collected using ResearchKit. *Sci Data.* 2016;3(1):160011. 10.1038/sdata.2016.11 26938265 PMC4776701

[8] Chan YY Wang P Rogers L : The Asthma Mobile Health Study, a large-scale clinical observational study using ResearchKit. *Nat Biotechnol.* 2017;35(4):354–362. 10.1038/nbt.3826 28288104 PMC5559298

[9] Deering S Pratap A Suver C : Real-world longitudinal data collected from the SleepHealth mobile app study. *Sci Data.* 2020;7(1):418. 10.1038/s41597-020-00753-2 33247114 PMC7695828

[10] Omberg L Chaibub Neto E Perumal TM : Remote smartphone monitoring of Parkinson's disease and individual response to therapy. *Nat Biotechnol.* 2021;9:1–8. 10.1038/s41587-021-00974-9 34373643 PMC12812035

[11] Pratap A Grant D Vegesna A : Evaluating the Utility of Smartphone-Based Sensor Assessments in Persons With Multiple Sclerosis in the Real-World Using an App (elevateMS): Observational, Prospective Pilot Digital Health Study. *JMIR Mhealth Uhealth.* 2020;8(10):e22108. 10.2196/22108 33107827 PMC7655470

[12] Renn BN Pratap A Atkins DC : Smartphone-based passive assessment of mobility in depression: Challenges and opportunities. *Ment Health Phys Act.* 2018;14:136–139. 10.1016/j.mhpa.2018.04.003 30123324 PMC6095666

[13] Pratap A Neto EC Snyder P : Indicators of retention in remote digital health studies: a cross-study evaluation of 100,000 participants. *NPJ Digit Med.* 2020;3(1):21. 10.1038/s41746-020-0224-8 32128451 PMC7026051

[14] Mangravite LM Sen A Wilbanks JT : Mechanisms to Govern Responsible Sharing of Open Data: A Progress Report.Manubot;2020; Accessed August 13, 2021. Reference Source

[15] Holzer JK Ellis L Merritt MW : Why We Need Community Engagement in Medical Research. *J Investig Med.* 2014;62(6):851–855. 10.1097/JIM.0000000000000097 24979468 PMC4108547

[16] 26 ingredients to beat youth anxiety & depression: reviewing the evidence. Accessed July 30, 2021. Reference Source

[17] McWhirter RE Critchley CR Nicol D : Community Engagement for Big Epidemiology: Deliberative Democracy as a Tool. *J Pers Med.* 2014;4(4):459–474. 10.3390/jpm4040459 25563457 PMC4282883

[18] Israel BA Schulz AJ Parker EA : Community-based participatory research: policy recommendations for promoting a partnership approach in health research. *Educ Health (Abingdon).* 2001;14(2):182–197. 10.1080/13576280110051055 14742017

[19] Byrne L Wykes T : A role for lived experience mental health leadership in the age of Covid-19. *J Ment Health.* 2020;29(3):243–246. 10.1080/09638237.2020.1766002 32449392

[20] Hawke LD Relihan J Miller J : Engaging youth in research planning, design and execution: Practical recommendations for researchers. *Health Expect.* 2018;21(6):944–949. 10.1111/hex.12795 29858526 PMC6250868

[21] Honey A Boydell KM Coniglio F : Lived experience research as a resource for recovery: a mixed methods study. *BMC Psychiatry.* 2020;20(1):456. 10.1186/s12888-020-02861-0 32958045 PMC7507671

[22] The MindKind Consortium: MindKind 2021 MindKind DataBank: Appendix 1 (Version V1.0). *Synapse.* 2021a. 10.7303/syn26067677

[23] The MindKind Consortium: MindKind 2021 MindKind DataBank: Appendix 3 (Version V1.0). *Synapse.* 2021c. 10.7303/syn26067679.1

[24] USDA Food Insecurity - Six Item.2012; Accessed July 29, 2021. Reference Source

[25] Hamilton CM Strader LC Pratt JG : The PhenX Toolkit: get the most from your measures. *Am J Epidemiol.* 2011;174(3):253–260. 10.1093/aje/kwr193 21749974 PMC3141081

[26] Organization WH: Measuring Health and Disability: Manual for WHO Disability Assessment Schedule WHODAS 2.0.World Health Organization;2010. Reference Source

[27] Kroenke K Spitzer RL Williams JB : The PHQ-9: validity of a brief depression severity measure. *J Gen Intern Med.* 2001;16(9):606–613. 10.1046/j.1525-1497.2001.016009606.x 11556941 PMC1495268

[28] Spitzer RL Kroenke K Williams JB : A brief measure for assessing generalized anxiety disorder: the GAD-7. *Arch Intern Med.* 2006;166(10):1092–1097. 10.1001/archinte.166.10.1092 16717171

[29] The MindKind Consortium: MindKind 2021 MindKind DataBank: Appendix 2 (Version V1.0). *Synapse.* 2021b. 10.7303/syn26067678.1

[30] Morin CM Belleville G Bélanger L : The Insomnia Severity Index: Psychometric Indicators to Detect Insomnia Cases and Evaluate Treatment Response. *Sleep.* 2011;34(5):601–608. 10.1093/sleep/34.5.601 21532953 PMC3079939

[31] Hughes ME Waite LJ Hawkley LC : A Short Scale for Measuring Loneliness in Large Surveys: Results From Two Population-Based Studies. *Res Aging.* 2004;26(6):655–672. 10.1177/0164027504268574 18504506 PMC2394670

[32] Craig CL Marshall AL Sjöström M : International Physical Activity Questionnaire: 12-Country Reliability and Validity. *Med Sci Sports Exerc.* 2003;35(8):1381–1395. 10.1249/01.MSS.0000078924.61453.FB 12900694

[33] International Physical Activity Questionnaire.Accessed July 29, 2021. Reference Source

[34] Kanter JW Mulick PS Busch AM : The Behavioral Activation for Depression Scale (BADS): Psychometric Properties and Factor Structure. *J Psychopathol Behav Assess.* 2007;29(3):191. 10.1007/s10862-006-9038-5

[35] Derry JMJ Mangravite LM Suver C : Developing predictive molecular maps of human disease through community-based modeling. *Nat Genet.* 2012;44(2):127–130. 10.1038/ng.1089 22281773 PMC3643818

[36] Morain SR Whicher DM Kass NE : Deliberative Engagement Methods for Patient-Centered Outcomes Research. *Patient.* 2017;10(5):545–552. 10.1007/s40271-017-0238-8 28374286

[37] De Vries R Stanczyk A Wall IF : Assessing the quality of democratic deliberation: A case study of public deliberation on the ethics of surrogate consent for research. *Soc Sci Med.* 2010;70(12):1896–1903. 10.1016/j.socscimed.2010.02.031 20378225 PMC2866810

[38] O’Doherty KC Burgess MM : Engaging the Public on Biobanks: Outcomes of the BC Biobank Deliberation. *Public Health Genomics.* 2009;12(4):203–215. 10.1159/000167801 19367089

[39] Gale NK Heath G Cameron E : Using the framework method for the analysis of qualitative data in multi-disciplinary health research. *BMC Med Res Methodol.* 2013;13(1):117. 10.1186/1471-2288-13-117 24047204 PMC3848812

