# Supplementary material for: MindKind: A mixed-methods protocol for the feasibility of global digital mental health studies in young people
Source: Wellcome Open Res. 2022 May 12;6:275. Originally published 2021 Oct 15. [Version 2] doi: 10.12688/wellcomeopenres.17167.2 (PMC9160707; doi:10.12688/wellcomeopenres.17167.2)
Supplement: Peer review history

# Peer review: Reviewer response for version 2

**DOI:** 10.21956/wellcomeopenres.19807.r50493

**Published:** 2022-06-01

**Authors:** Rahul Shidhaye

**Affiliations:** 1 Pravara Institute of Medical Sciences, Loni, Maharashtra, India

Many thanks for addressing all the concerns and revising the manuscript.

Is the study design appropriate for the research question?

Yes

Is the rationale for, and objectives of, the study clearly described?

Yes

Are sufficient details of the methods provided to allow replication by others?

Yes

Are the datasets clearly presented in a useable and accessible format?

Not applicable

Reviewer Expertise:

Global Mental Health, Implementation Research, Yoga and Mental Health

I confirm that I have read this submission and believe that I have an appropriate level of expertise to confirm that it is of an acceptable scientific standard.

# Peer review: Reviewer response for version 1

**DOI:** 10.21956/wellcomeopenres.18962.r46498

**Published:** 2022-01-18

**Authors:** Manasi Kumar

**Affiliations:** 1 Department of Psychiatry, University of Nairobi, Nairobi, Kenya

While I find the overall study very insightful and compelling, the language of the paper was a bit concerning. The feasibility study is embedded in a mixed-methods framing of a pilot 12-week app-based sub-study nested within the willingness to engage as well as understanding the preferences of youth. It is in both these domains that I find the study lacking. It appears very dominated by trial language and the process indicators and process as an ethical ontological and phenomenological problem hasn't been teased out. I find the use of the option 'democracy' limiting and mislabelled. Does the conceptual basis of democracy imply greater access to the use of data or say in the generation of data? Could the frame of reference for acceptability be improved?

 Ultimately, how issues of power, access to information provided to secure funding, and advancing advocacy and research, addressing data protection and data security from national/LMIC/youth perspectives, I am not convinced that quantitative substudy needs a study design section for the governance model. More clarity is needed here.

 My general feedback is that while the overall questions on choices are highly regimented, I want to know if these are ideas that were generated by the various consultations or will these be tweaked as further advisory boards and consultations are carried out.

Is the study design appropriate for the research question?

Partly

Is the rationale for, and objectives of, the study clearly described?

Partly

Are sufficient details of the methods provided to allow replication by others?

Partly

Are the datasets clearly presented in a useable and accessible format?

Not applicable

Reviewer Expertise:

Global mental health, youth mental health, global health partnerships and research

I confirm that I have read this submission and believe that I have an appropriate level of expertise to confirm that it is of an acceptable scientific standard, however I have significant reservations, as outlined above.

# Response

**Published:** 2022-05-02

**Authors:** Solveig Sieberts

**Affiliations:** Sage Bionetworks, USA

- While I find the overall study very insightful and compelling, the language of the paper was a bit concerning. The feasibility study is embedded in a mixed-methods framing of a pilot 12-week app-based sub-study nested within the willingness to engage as well as understanding the preferences of youth. It is in both these domains that I find the study lacking. It appears very dominated by trial language and the process indicators and process as an ethical ontological and phenomenological problem hasn't been teased out. I find the use of the option 'democracy' limiting and mislabelled. Does the conceptual basis of democracy imply greater access to the use of data or say in the generation of data? Could the frame of reference for acceptability be improved?

**Response: **Within the MindKind app, there are four data governance arms to which participants are randomized, one of which is labeled within *this paper* as “democracy” because each participant has equal say in choosing the terms of data sharing. However, *within the app itself*, we do not use the term “democracy”; youth participants are not exposed to this potentially biasing term. This said, we do use the term “vote” as we do ask participants to vote on their preferred data governance choices. For example, the language for the Option D (Democracy + Server Access) is as follows: “You and other participants get to vote how researchers can access and use your data. Researchers will only view your data on a secure server - they cannot download your data.  Before researchers can use the data, we will tell you the voting results.”

- Ultimately, how issues of power, access to information provided to secure funding, and advancing advocacy and research, addressing data protection and data security from national/LMIC/youth perspectives, I am not convinced that quantitative substudy needs a study design section for the governance model. More clarity is needed here.

**​​​​​​​Response: **There have been no studies to date that have directly examined a multinational cohort of young peoples’ preferences with regard to big health data governance. In this study, we approach assessing young peoples’ preferences for data governance both through qualitative (deliberative democracy) and quantitative (via our pilot app) methods. Our vision is that these two data streams together will provide powerful insights: from the deliberative democracy sessions understanding of young peoples’ perception of, feelings and opinions toward, and preferences with regards to data governance, as well as how this grounding translates (or doesn’t) into big health data research participation as captured by the pilot app. We have added Table 2 to better highlight the information gained from each substudy with respect to data governance.   

- My general feedback is that while the overall questions on choices are highly regimented, I want to know if these are ideas that were generated by the various consultations or will these be tweaked as further advisory boards and consultations are carried out.

**​​​​​​​Response: **Youth leads and youth panel members were involved in the study design and development of data collection tools from early on in protocol development. We elicited their feedback in the survey questions about active ingredients and mental health indicators, the data governance models for the randomized study arms, and the qualitative study design. All procedures described in this protocol manuscript are inclusive of youth lead and youth panel feedback. We will likely not make further modifications to our questionnaires or study design, but will continue to have ongoing meetings with youth leads and youth panel members and will be flexible in incorporating any perspectives on revised procedures as our resources and timelines allow. Table 2 outlines the roles played by the advisory boards in the final designs.

# Peer review: Reviewer response for version 1

**DOI:** 10.21956/wellcomeopenres.18962.r47159

**Published:** 2022-01-10

**Authors:** Keng-Yen Huang

**Affiliations:** 1 Department of Population Health, New York University Grossman School of Medicine, New York, NY, USA

The protocol paper aims to describe the MindKind study, a technology approach to mental health intervention for young people. This protocol focuses on understanding the data governance and engagement strategies influencing young peoples’ (aged 16-24) participation in app-based mental health promotion/studies. This study applies mixed methods and consists of two substudies.

 Overall, this paper has several strengths. It is timely and targets several important research needs in applying technology for young people’s mental health promotion. Conducting the study in three countries and utilizing a global research network (including young people from multiple countries) are innovated. However, the writing is not always clear. The paper can be improved by better organization and better defining research questions. The research questions proposed to be studied are not always clear (e.g., questions are presented differently in different sections), and some information presented may not be in the right section. Below I list suggestions for improving organization/readability/clarity.

 INTRODUCTION.

- The fourth paragraph is confusing. The paragraph starts with describing methods and hypotheses before describing the MindKind Project and its aims. It will be much clearer if the order is reversed. The authors should also clarify whether the protocol paper’s aims are the same as the MindKind Project Aims. The goals/aims presented in several sections don’t seem to be the same.   
- To me, it seems that MindKind is to study two areas of questions related to technology use in young people’s mental health promotion: (1) One is to study engagement strategies for mental health app development. Through a purpose-built app, the team will study the feasibility and acceptability of the app approach/strategies (using a mixed-method design), as well as understand potential influential factors and mechanisms. (2) The other is to study data collection/governing/sharing models (i.e., to identify acceptable approaches for young people to report their mental health data/contribute to databank using app data collection; to understand young people’s preferred ways for governance/sharing their data with research scientists).  
- The MindKind mental health app considers 5 active ingredients (sleep, body movement, social connections, positive activities). It will be helpful to introduce the framework/concept in the introduction as part of the MindKind overview first (rather than describe this in the method and mix with other design info), and then introduce this protocol will test an App engagement approach (by studying whether given-option-to-choose would be more acceptable than the fix-sequence approach using an RCT/quantitative design).  
- MindKind is guided by the Youth Engagement/partnership approach. It might be helpful to briefly describe this approach as part of the MindKind project overview in the Introduction section (e.g., moving the descriptions from method to introduction & using a figure to present the structure).  
- I would also suggest moving detailed descriptions about the youth group & research structure in the appendix. The current presentation format is a bit distractive because of too much info & not fully integrated with the two substudies.   

METHODS.

- For clarity, it will be helpful to organize the method section by research questions (engagement strategies for app design vs. data governance) rather than by types of study (qualitative vs quantitative). The current presentation approach is a bit confusing. When reading each substudy, it’s not quite clear what research questions the team is trying to study. For a research paper, it tends to be easier to understand if the goals/objectives are presented first, followed by presenting methods used to answer the questions.  
- Please clearly state the research questions at the beginning of each substudy section, and describe data collection methods/measures separately for each research question. There are several research questions studied in each substudy, but the method for each research question is not clear. Using a table can help.   
- For each substudy, please clarify how youth-researchers (or different youth group structures) will participate in each substudy (e.g., do they help design, recruit, analyze data). How are they working with the expert researchers?

- What is the age range of the adolescents to be studied?
- Figure 2. There are 4 active ingredient areas, but intervention arm 2 only includes 3 ingredients. Why?
- The study timeline is not clear. Would quantitative or qualitative studies be conducted simultaneously or sequentially? Would quantitative data be used to inform the qualitative study?
- Figure 3 is a very intense data collection procedure. Is this part of the co-design with the youth? Would this be modified if the youths do not think this approach will work?

Is the study design appropriate for the research question?

Partly

Is the rationale for, and objectives of, the study clearly described?

Partly

Are sufficient details of the methods provided to allow replication by others?

Partly

Are the datasets clearly presented in a useable and accessible format?

Partly

Reviewer Expertise:

Child and adolescent mental health research (epidemiology, mHealth intervention development, and testing, intervention implementation/user-center testing)

I confirm that I have read this submission and believe that I have an appropriate level of expertise to confirm that it is of an acceptable scientific standard, however I have significant reservations, as outlined above.

# Response

**Published:** 2022-05-02

**Authors:** Solveig Sieberts

**Affiliations:** Sage Bionetworks, USA

The protocol paper aims to describe the MindKind study, a technology approach to mental health intervention for young people. This protocol focuses on understanding the data governance and engagement strategies influencing young peoples’ (aged 16-24) participation in app-based mental health promotion/studies. This study applies mixed methods and consists of two substudies.

 Overall, this paper has several strengths. It is timely and targets several important research needs in applying technology for young people’s mental health promotion. Conducting the study in three countries and utilizing a global research network (including young people from multiple countries) are innovated. However, the writing is not always clear. The paper can be improved by better organization and better defining research questions. The research questions proposed to be studied are not always clear (e.g., questions are presented differently in different sections), and some information presented may not be in the right section. Below I list suggestions for improving organization/readability/clarity.

 INTRODUCTION.

- The fourth paragraph is confusing. The paragraph starts with describing methods and hypotheses before describing the MindKind Project and its aims. It will be much clearer if the order is reversed. The authors should also clarify whether the protocol paper’s aims are the same as the MindKind Project Aims. The goals/aims presented in several sections don’t seem to be the same.  

**Response: **Thank you for this observation. We have removed the discussion of the overarching program aims and focused more on the specific aims of the (sub)studies described here. We have re-written this paragraph and clarified the study aims there, in the Study Overview section as well as the individual substudy sections per your suggestions that follow.

- To me, it seems that MindKind is to study two areas of questions related to technology use in young people’s mental health promotion: (1) One is to study engagement strategies for mental health app development. Through a purpose-built app, the team will study the feasibility and acceptability of the app approach/strategies (using a mixed-method design), as well as understand potential influential factors and mechanisms. (2) The other is to study data collection/governing/sharing models (i.e., to identify acceptable approaches for young people to report their mental health data/contribute to databank using app data collection; to understand young people’s preferred ways for governance/sharing their data with research scientists). 

**​​​​​​​Response: **See response above.

- The MindKind mental health app considers 5 active ingredients (sleep, body movement, social connections, positive activities). It will be helpful to introduce the framework/concept in the introduction as part of the MindKind overview first (rather than describe this in the method and mix with other design info), and then introduce this protocol will test an App engagement approach (by studying whether given-option-to-choose would be more acceptable than the fix-sequence approach using an RCT/quantitative design). 

**​​​​​​​Response: **While the AIs are an integral part of the participants’ experience in the app, they are a secondary concept relative to the overall aims, and only specifically related to the quantitative substudy. As such, we believe that this information would actually detract from the message of the introduction, rather than add to it. We have improved the description of the four AIs in the *Study Overview* section. 

- MindKind is guided by the Youth Engagement/partnership approach. It might be helpful to briefly describe this approach as part of the MindKind project overview in the Introduction section (e.g., moving the descriptions from method to introduction & using a figure to present the structure). 

**​​​​​​​Response: **Thank you for this suggestion. We have added these details to the introduction section, as follows:

 “Throughout the project we centre our work on the voices of young people, by grounding our work in a youth-adult participatory research approach whereby we seek to involve youth stakeholders and researchers as equal partners. There are several ways for young people with lived experiences of mental health issues to be involved in our MindKind work, including as youth leads who are employed as members of the study staff, as in-country youth panel members, as multi-national youth panel members, and as research participants.”

- I would also suggest moving detailed descriptions about the youth group & research structure in the appendix. The current presentation format is a bit distractive because of too much info & not fully integrated with the two substudies.  

**​​​​​​​Response: **While we agree that this information is not common in protocol papers such as these, we feel that this is an important and somewhat novel feature of our project and study methodology that would be minimized by relegating it to an appendix stored in an external data repository. We have substantially shortened the descriptions in the text and moved this information to Table 1. We believe that this compromise improves the readability of the paper while maintaining the focus on youth involvement.

 METHODS.

- For clarity, it will be helpful to organize the method section by research questions (engagement strategies for app design vs. data governance) rather than by types of study (qualitative vs quantitative). The current presentation approach is a bit confusing. When reading each substudy, it’s not quite clear what research questions the team is trying to study. For a research paper, it tends to be easier to understand if the goals/objectives are presented first, followed by presenting methods used to answer the questions. 

**​​​​​​​Response: **We respectfully disagree with your suggestion that the paper clarity would be improved by organizing the paper by question versus substudy. We have added language at the beginning of each substudy section, reintroducing the substudy aims as well as added a table (Table 2) describing the research questions, and how they’re addressed by each of the substudies. We hope this will serve as a guidepost to help readers better understand the relationship between the substudies.

- Please clearly state the research questions at the beginning of each substudy section, and describe data collection methods/measures separately for each research question. There are several research questions studied in each substudy, but the method for each research question is not clear. Using a table can help.  

**​​​​​​​Response: **Thank you for this suggestion. As noted above, we have added a table describing the study questions and how they are addressed in each substudy, as well as reintroducing the substudy aims in each section.

- For each substudy, please clarify how youth-researchers (or different youth group structures) will participate in each substudy (e.g., do they help design, recruit, analyze data). How are they working with the expert researchers?

**​​​​​​​Response: **We have included this information in Table 2.

- What is the age range of the adolescents to be studied?

**​​​​​​​Response: **This is outlined in the first paragraph of the *MindKind Study Design* section and pertains to both the Quantitative and Qualitative substudies:

 “To participate in the MindKind study, youth participants must live in one of the participating countries and be legally able to provide consent (age 16–24 in the UK or 18–24 for India and South Africa).“

- Figure 2. There are 4 active ingredient areas, but intervention arm 2 only includes 3 ingredients. Why?

**​​​​​​​Response: **The study consists of 3 4-week rotations on a single topic (AI), so participants will not participate in all AIs regardless of which arm they are in. We have amended the figure captions to try to provide more clarity:

 “In this example, the ARM 1 participant has *selected* the topics Body movement, Positive activities and Social connections for their topics, while the ARM 2 participant has been *randomly assigned* to Sleep, Positive activities and Body movement for their 3 topics.”

- The study timeline is not clear. Would quantitative or qualitative studies be conducted simultaneously or sequentially? Would quantitative data be used to inform the qualitative study?

**​​​​​​​Response: **Both substudies are being conducted simultaneously as two-pronged approaches to answering the questions about youths’ feelings, preferences, and concerns about data governance structures for sharing their mental health data, as well as their willingness to participate. We will synthesize the results from both substudies to form our conclusions. It is also important to note that the choice of governance models used in the quantitative study have been selected based on feedback from the youth advisory panels, so have already been informed by the voices of the youth. We have added the following text for clarity:

 “The two substudies will run concurrently, and results from the two will be compared to understand participants’ ideals and their effect on participation relative to current research standards.”

- Figure 3 is a very intense data collection procedure. Is this part of the co-design with the youth? Would this be modified if the youths do not think this approach will work?

**​​​​​​​Response: **The data collection process described in Figure 3 was developed in collaboration with the professional youth advisors and youth advisory panels. We presented three different scenarios to the youth groups and incorporated their feedback into the design of the survey. This survey cadence presented in Figure 3 was deemed the least burdensome. Youth groups also informed strategies to maximize engagement with surveys, such as reflection exercises and GIFs for performing surveys. These strategies have also been used successfully in other studies (see Nickels *et al* cited below*).

 The survey approach consisted of a brief daily survey that asked one question about mood and 1 to 5 questions about the active ingredient to be studied. It also included one weekly survey of mood standard instruments as well as the selected or assigned active ingredient. We timed the length of each survey type and found that on average daily surveys took no more than 5 minutes to complete in total, with a range of 30 secs to 5 minutes, with the weekly survey taking an average of 9 minutes, with a range of 3-22 minutes.

 The last week of the study consists of a remote user survey to determine what aspects of the experiment were burdensome, which engagement strategies were deemed helpful in motivating youth to complete the survey, and ideas from youth to improve the survey experience. A similar survey is also administered when a participant asks to leave the study or has not completed a survey after 1 week. This information will be used to improve the survey experience.

 We have added a brief statement describing these efforts in the manuscript.

 * Nickels S, Edwards MD, Poole SF, Winter D, Gronsbell J, Rozenkrants B, Miller DP, Fleck M, McLean A, Peterson B, Chen Y, Hwang A, Rust-Smith D, Brant A, Campbell A, Chen C, Walter C, Arean PA, Hsin H, Myers LJ, Marks WJ Jr, Mega JL, Schlosser DA, Conrad AJ, Califf RM, Fromer M. Toward a Mobile Platform for Real-world Digital Measurement of Depression: User-Centered Design, Data Quality, and Behavioral and Clinical Modeling. JMIR Ment Health. 2021 Aug 10;8(8):e27589. doi: 10.2196/27589. PMID: 34383685; PMCID: PMC8386379.

# Peer review: Reviewer response for version 1

**DOI:** 10.21956/wellcomeopenres.18962.r46500

**Published:** 2021-12-21

**Authors:** Rahul Shidhaye

**Affiliations:** 1 Pravara Institute of Medical Sciences, Loni, Maharashtra, India

This study protocol by the MindKind consortium describes a mixed-methods approach to explore the data governance models that will influence the engagement of young people in mobile app-based mental health studies. This is a fairly well-written protocol. There are a few concerns which the authors may wish to address in the next version.

1. Participants will be randomized to one of the two engagement strategies as illustrated in Figure 2. Are there any other strategies as well to engage participants and improve compliance? This is important to know as the engagement with the mobile app will be determined by a multitude of factors. One of these is the way in which the modules are presented, and the other is the data governance structure. What other factors which affect engagement and compliance are measured in this study? One example is user interface and user experience. There could be multiple other factors that can be important confounders. Please clarify.
2. On page 10, paragraph on governance acceptability, it is mentioned that the effect of the governance model on study enrolment will be assessed. It will also be good to study the effect of the governance model on engagement and compliance. This is probably mentioned in the next paragraph. Please confirm.
3. Will the participants randomized to Group B-D be aware of different types of data governance structures? Or will they be randomly allocated to the group first and then they will be only informed about the allocation? This will have important implications in terms of their enrolment and engagement.
4. Baseline awareness about the rights of an individual on their personal data will play an important role in determining the extent to which participants contribute to deliberate democracy approach. Hopefully, the educational materials will cover this aspect and provide information to the participants.
5. How challenging is it to organize deliberative democracy sessions as an online group?
6. Is there a plan to triangulate the data from the two approaches? If yes, it will be good to add a short description about the same.
7. Please describe how the data gathered in the study will be protected.
8. Professional Youth Advisors will be full-time hired by the project. Similarly, the members of the Youth Panel Advisory Groups and International Youth Panel members will also be hired full-time? If not, will they receive any honorarium for their participation? Participants in the qualitative sub-study will also spend a good amount of their time - will they be remunerated for the same?

Is the study design appropriate for the research question?

Yes

Is the rationale for, and objectives of, the study clearly described?

Yes

Are sufficient details of the methods provided to allow replication by others?

Yes

Are the datasets clearly presented in a useable and accessible format?

Not applicable

Reviewer Expertise:

Global Mental Health, Implementation Research, Yoga and Mental Health

I confirm that I have read this submission and believe that I have an appropriate level of expertise to confirm that it is of an acceptable scientific standard, however I have significant reservations, as outlined above.

# Response

**Published:** 2022-05-02

**Authors:** Solveig Sieberts

**Affiliations:** Sage Bionetworks, USA

This study protocol by the MindKind consortium describes a mixed-methods approach to explore the data governance models that will influence the engagement of young people in mobile app-based mental health studies. This is a fairly well-written protocol. There are a few concerns which the authors may wish to address in the next version.

- 1. Participants will be randomized to one of the two engagement strategies as illustrated in Figure 2. Are there any other strategies as well to engage participants and improve compliance? This is important to know as the engagement with the mobile app will be determined by a multitude of factors. One of these is the way in which the modules are presented, and the other is the data governance structure. What other factors which affect engagement and compliance are measured in this study? One example is user interface and user experience. There could be multiple other factors that can be important confounders. Please clarify.

**Response: **For the purposes of this study, we are only perturbing data governance and selection of AIs vs fixed experience. You are correct that there are a number of factors that may improve governance, but these are not explored exhaustively here. We would love to do that in the future, but it was not feasible to do so in this pilot. We have added a paragraph to the *Limitations* section of the Discussion to this effect:

 “Additionally, the engagement question posed in this study is limited to the effects of AI choice and governance model on participation. This is not a thorough study of optimal app-based engagement strategies. While we have incorporated youth feedback with respect to governance models, AI topics, survey and study design, website and app designs, and user interfaces, a more iterative user-centered design approach should be incorporated in future iterations of the study. Additionally, we have not explored other engagement strategies such as in-app or text message notifications, gamification or other methods of increasing engagement, though these would be important factors to consider in future efforts.”

- 2. On page 10, paragraph on governance acceptability, it is mentioned that the effect of the governance model on study enrolment will be assessed. It will also be good to study the effect of the governance model on engagement and compliance. This is probably mentioned in the next paragraph. Please confirm.

**Response: **Thank you for pointing this out. While this is mentioned in the *Data Analysis Plan* for Engagement, this is easily overlooked. We have added a sentence at the end of the *Study Design: governance* section to highlight this intention:

 “We will also assess the effect of governance models on study engagement and retention (See the *Data Analysis Plan* Engagement section for details).”

- 3. Will the participants randomized to Group B-D be aware of different types of data governance structures? Or will they be randomly allocated to the group first and then they will be only informed about the allocation? This will have important implications in terms of their enrolment and engagement.

**​​​​​​​Response: **Participants in B-D will have no knowledge of other potential governance structures. They will be presented with an informed consent outlining the data governance for the model to which they were randomized, without mention of other alternatives. In this way, we mirror the experience they would have in a typical research study. We have clarified this in the text:

 “In order to mirror the experience in a typical study, participants will be exposed to an informed consent specific to their data governance model and can choose to either join or not. They will have no exposure to other potential governance models.”

- 4. Baseline awareness about the rights of an individual on their personal data will play an important role in determining the extent to which participants contribute to deliberate democracy approach. Hopefully, the educational materials will cover this aspect and provide information to the participants.

**​​​​​​​Response: **Prior to their first deliberative democracy session, participants are instructed to watch two video modules that contain comprehensive, engaging information about different models of data governance used to organize research data, including addressing the rights of individuals. A formal manuscript describing the educational materials in detail will be forthcoming.

- 5. How challenging is it to organize deliberative democracy sessions as an online group?

**​​​​​​​Response: **Organizing deliberative democracy sessions online has posed a number of challenges and opportunities. Briefly, pragmatic challenges include multinational scheduling, the length of online sessions (reasonable attention spans), and technology access issues. Conceptual challenges include educational provision and the roles of facilitators. Given that this is a protocol paper, we don’t feel it appropriate to add these retrospective learnings here, however, we will discuss these challenges and opportunities further in our forthcoming analysis paper.

- 6. Is there a plan to triangulate the data from the two approaches? If yes, it will be good to add a short description about the same.

**​​​​​​​Response: **Yes, we have added the following sentence to the “Interplay between quantitative and qualitative studies” section:

 “Results from the two substudies will be compared to understand participants’ ideals and their effect on participation relative to current research standards.”

- 7. Please describe how the data gathered in the study will be protected.

**​​​​​​​Response: **We added a *Data Protection* section in both the Quantitative and Qualitative Study sections to describe the security and privacy measures to safeguard the confidentiality and integrity of the data and prevent improper access.

- 8. Professional Youth Advisors will be full-time hired by the project. Similarly, the members of the Youth Panel Advisory Groups and International Youth Panel members will also be hired full-time? If not, will they receive any honorarium for their participation? Participants in the qualitative sub-study will also spend a good amount of their time - will they be remunerated for the same?

**​​​​​​​Response: **Youth panel members participate in two meetings monthly + occasional asynchronous assignments. This information has been added to the newly added Table 1. Members are either paid an honorarium or hourly, depending on the site.

 Qualitative substudy participants receive incentives for participation in deliberative democracy sessions. In the UK, participants were offered a 30GBB gift card for each session in which they participated (maximum compensation 60GBP). In India, participants were offered a single 500INR gift card for all deliberative session participation (maximum compensation 500INR). In South Africa, deliberative session participants received a 400ZAR airtime voucher per session (maximum compensation 800ZAR). Additionally, South African participants of the quantitative study received a 150ZAR airtime voucher per month of participation. This information has been added as follows:

 “Given the amount of time required for pre-learning activities and participation, qualitative substudy participants will receive a modest incentive for participation in the form of a gift card (UK, India) or airtime voucher (South Africa).”
